# 

**DOI:** 10.3325/cmj.2014.55.78b

**Published:** 2014-02

**Authors:** Editorial team of the Croatian Medical Journal

In Reply: The editorial team of the *Croatian Medical Journal* wishes to express regret about inappropriate selection of terms in the case report by Nosan et al (1). We welcome the discussion and agree with Dr Masukume that the use of pejorative terms, including “antimongoloid” should be abandoned (2). We have also given the authors of the case report an opportunity to respond and express their opinion on this matter (3). From authors to publishers, all parties included in the scientific production should have their role in changing such practice. Using Down syndrome as an example, history shows us that usage of pejorative terms in scholarly articles can be altered for the better (4). The inappropriate term usage from the last issue of the *CMJ* reminds us that our ethical standards are in need of perpetual refinement, not only due to technological advancement, but also due to wider social and cultural changes. Although at first glance it may seem as a “technical“ issue, it is not by chance that fellow authors point to the fact that there could be more to term usage than simple denomination, and propose changes to already established terms (5). In the words of the great Boris Pasternak “...to call each thing by its right name“ has a value in itself. It is thus our moral obligation to constantly remind of and take action against inappropriate usage of discriminative and politically incorrect terms.
